# Use of Artificial Intelligence on Imaging and Preoperatory Planning of the Knee Joint: A Scoping Review

**DOI:** 10.3390/medicina61040737

**Published:** 2025-04-16

**Authors:** Luca Bertolino, Marta Bianca Maria Ranzini, Alberto Favaro, Elena Bardi, Flavio Lorenzo Ronzoni, Tommaso Bonanzinga

**Affiliations:** 1 IRCCS Humanitas Research Hospital, Via Manzoni 56, Rozzano, 20089 Milan, Italy; luca.bertolino@st.hunimed.eu (L.B.); marta.ranzini@humanitas.it (M.B.M.R.); alberto.favaro@humanitas.it (A.F.); elena.bardi@humanitas.it (E.B.); 2 Department of Biomedical Sciences, Humanitas University, Via Rita Levi Montalcini 4, Pieve Emanuele, 20072 Milan, Italy; flavio.ronzoni@hunimed.eu

**Keywords:** artificial intelligence, machine learning, deep learning, orthopedics, imaging, diagnostics, preoperative planning

## Abstract

*Background and Objectives*: This scoping review explores the current state of the art of AI-based applications in the field of orthopedics, focusing on its implementation in diagnostic imaging and preoperative planning of knee joint procedures. *Materials and Methods*: The search was carried out using the recognized scholarly databases PubMed, Medline and Embase and was set to identify original research addressing AI applied to imaging in knee diagnosis and surgical planning, written in English and published up to January 2025. *Results*: The search produced 1612 papers, of which 36 were included in our review. All papers addressed AI applied to common imaging methods in clinical practice. Of these, thirty integrated AI-based tools with X-rays, one applied AI to X-rays to produce CT-like 3D reproductions, and two studies applied AI to MRI. *Conclusions:* Several AI tools have already been validated for enhancing the accuracy of measurements and detecting additional parameters in a shorter time compared to standard assessments. We expect these may soon be introduced into routine clinical practice to streamline a number of technical tasks and in some cases to replace the need for human intervention.

## 1. Introduction

Artificial intelligence (AI) is a broad and rapidly evolving field of computer science focused on the design and development of intelligent machines able to perform tasks that typically require human intelligence skills, including learning, reasoning, problem-solving and perception [1]. Within the field of AI, the greatest achievements and applications have been through data-driven approaches such as machine learning (ML) and deep learning (DL). These approaches involve developing algorithms able to ingest considerable amounts of data to discover and model patterns and relationships within these data directly, learning from mistakes and improving their performance in a way that resembles experiential learning [1]. Specifically, ML aims to teach machines how to perform specific tasks by extracting representative features from the data and then identifying patterns from these features via mathematical models and algorithms. Deep learning is a subset of ML, which employs artificial neural networks (NNs), originally thought to mimic the human brain, to extract complex numerical features typically not directly correlated with human experience and perception [2,3]. This differs from traditional ML, where features are manually hand-crafted or selected (e.g., inclusion of clinical variables, use of radiomic features from imaging, etc.) and thus preserve a certain degree of interpretability [3]. NN architectures typically used in the imaging domain can be broadly classified into convolutional neural networks (CNNs) and Vision Transformers (ViT), the former excelling in analyzing local image features with filters, the latter able to more effectively capture global context and relationships thanks to the attention mechanisms [4]. Recurrent Neural Networks (RNNs) are also frequently adopted architectures for the analysis of time series, thus finding application in image sequences/video analysis as well as surgical planning pathways [4]. Overall, DL methods require larger data for model development and often lack interpretability compared to ML, but their predictive capabilities proved to be extremely powerful and well-performing on numerous tasks [3].

In orthopedic medical imaging, AI finds vast applicability especially for tasks such as imaging quality enhancement, image classification and detection and segmentation.

Indeed, AI tools enable users to improve image quality by reducing noise, improving the image resolution, optimizing contrast, making imaging more reliable for diagnosis and planning.

Other AI algorithms can classify images into categories, enabling fast and automated identification of pathological condition. Finally, AI-based tools can accurately detect and outline anatomical structures or pathological areas within the image (image detection and segmentation), favoring a quantitative assessment of regions of interest (e.g., by measuring surface/volume) as well as supporting surgical planning.

The automation of these tasks via AI can impact several aspects of the patient journey and support the healthcare professionals in providing superior quality of care. With specific reference to the orthopedics field, AI has shown promising results in improving diagnostic accuracy, supporting clinical decision-making, optimizing and personalizing surgical and/or treatment planning and monitoring patients’ outcome [2,5]. In 2020, a study by Federer et al. [5] provided an overview of such applications in orthopedics. Since then, a growing body of research has followed with numerous publications. Here, we provide an updated mapping of the literature since 2020, with a specific focus on developments and use of AI applications in diagnostic imaging and preoperative planning of knee joint procedures.

## 2. Materials and Methods

The review process was conducted according to the Preferred Reporting Items for Systematic Reviews and Meta-Analyses extension for scoping reviews (PRISMA-SC) [6].

The Population, Intervention, Comparator, and Outcomes of Interest enabled the formulation of the following broad research questions: How is AI being used in the analysis of images for knee diagnosis and knee surgery planning? Is AI an optimum tool for improving diagnostic accuracy, surgical outcomes, and postoperative assessments?

A systematic literature search was carried out using scholarly databases without the aid of AI tools. The search consisted of a single search of PubMed, Medline, and Embase accessed through IRCCS Humanitas Research Hospital. The queries searched for this scoping review were: (artificial intelligence OR machine learning OR deep learning) AND orthopedics AND knee AND (imaging OR preoperative planning). No searches were performed to explore dissemination material or gray literature.

Following the manual removal of duplicates, two reviewers (L.B., M.B.M.R.) independently screened the titles and abstracts using the eligibility criteria. The only inclusion criterion was work presented as an original research article investigating AI applications in diagnostic imaging or preoperative planning for knee surgery, while criteria for exclusion were formats other than original articles, thus reviews, case reports, letters/editorials, book chapters, methodological papers and papers written in languages other than English. The full texts of all eligible articles were retrieved and reviewed independently by the same two reviewers (L.B., M.B.M.R.) to assess their appropriateness to the research question. Disagreements were discussed with the senior author (T.B.) and resolved by consensus. Finally, references of selected articles were checked to identify titles missed upon screening. Any studies meeting the inclusion criterion were integrated into the final selection. 

The following data were extracted and recorded on an Excel sheet: imaging type, AI type, AI aim/application, and the main results. When needed, Excel sheet functions were used to create chart plots.

No methodological quality of the studies reviewed was assessed for this scoping review.

## 3. Results

### 3.1. Screening and Selection

**Figure 1:**
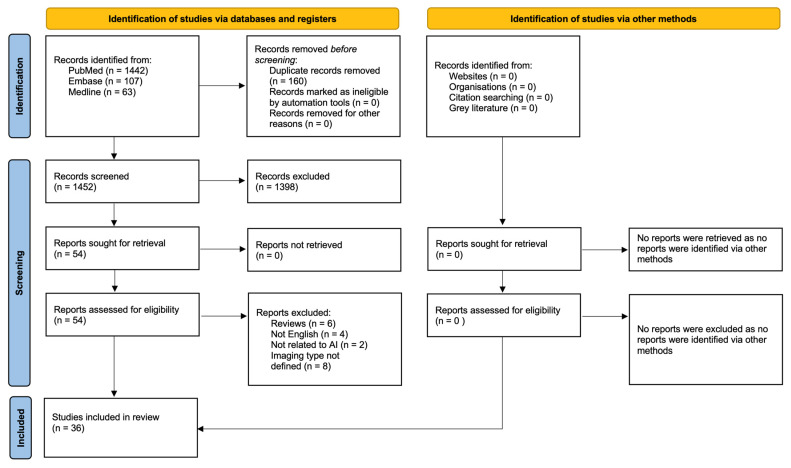
PRISMA 2020 flow diagram for new systematic reviews which included searches of databases, registers and other sources.

The systematic literature search yielded 1612 articles of which 160 were duplicates. In total, 1398 articles were excluded following title and abstract screening. The remaining 54 papers were all retrieved and later assessed according to the eligibility criteria, leaving 36 articles for review [7,8,9,10,11,12,13,14,15,16,17,18,19,20,21,22,23,24,25,26,27,28,29,30,31,32,33,34,35,36,37,38,39,40,41,42]. The screening and review process is summarized in Figure 1. No further studies were identified via other methods.

### 3.2. Overview of Papers

**Figure 2:**
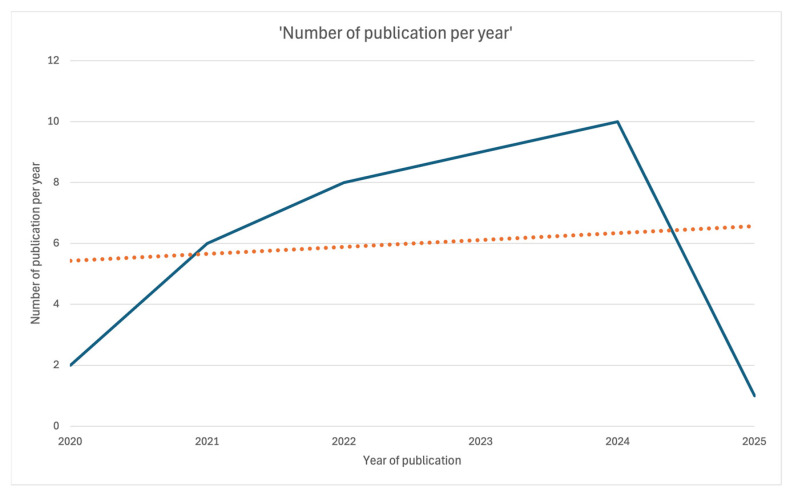
Linear plot showing the number of publications per year over time.

The present scoping review includes a total of 36 papers (Figure 1). All were published between 2020 and 2025: two in 2020 [7,21], six in 2021 [8,9,10,22,23,24], eight in 2022 [11,12,25,26,27,28,29,30], nine in 2023 [13,14,15,16,17,31,32,33,34], ten in 2024 [18,19,35,36,37,38,39,40,41,42], and one in January 2025 [25] (Figure 2).

The topics discussed in these papers included the type of AI technology, imaging technology, the operative setting and purposes for AI implementation. The articles reviewed and their main characteristics are summarized in Table 1 and Table 2, divided for ease of consultation according to their use in diagnostics or preoperative planning (Table 1 and Table 2).

### 3.3. Imaging Methods

All papers addressed AI applied to common imaging methods in clinical practice. The majority (n = 30) integrated AI-based tools with X-rays. Another one applied AI to X-rays to produce CT-like 3D reproductions [30]. Two studies applied AI to MRI [7,18]: one study employing a 5 min 3D quantitative double-echo steady-state (qDESS) sequence with automatic T2 mapping and deep learning super-resolution augmentation [7], the other applying automated femoral segmentation software to 3D high-resolution MRI to assess acute cartilage damage [18].

### 3.4. Range of AI Tool Applications

The AI tools in the papers reviewed were used to support operator assessments, either by enhancing imaging features or reducing operator-dependent factors (variability, misinterpretation) through automized assessments.

#### 3.4.1. OA Diagnosis

Several studies proposed ways for reducing variability among measurements for OA parameters, so as to allow more accurate evaluation of individual patient risk for OA progression. Two studies applied AI measuring quantitative cartilage parameters of knee osteoarthritis [12,19]. One study by Smolle et al. describes the use of AI-enhanced X-rays by means of the Knee Osteoarthritis Labeling Assistant (KOALA), a software providing both metric assessments of anterior–posterior (AP) or PA knee X-rays and proposals for clinical OA grade. The system has proven to markedly increase reader agreement rates for KL grade, sclerosis, JSN and osteophyte Osteoarthritis Research Society International (OARSI) grades both among senior readers and between senior and junior readers [12]. Brejnebøl et al. measured the improvements in assessing knee OA on radiographs according to the established KL grading system using the AI tool RBknee (version 2.1; Radiobotics). AI assistance increased junior readers’ radiographic KOA grading performance and increased interobserver agreement for osteoarthritis grading across radiologists and orthopedics of various experience levels [19].

#### 3.4.2. Fracture Detection

Another group of studies implemented ways for increasing fracture detections, especially difficult to identify from plain radiographs [8,9,10,18,30,33].

Three studies [8,9,10] explored AI-based technology to increase fracture recognition from X-rays. The 2021 study by Liu focused on the use of AI-based tools in the detection of tibial plateau fractures, reaching a level of accuracy comparable to a human operator—though at the time of the study, the tool was still unable to replace evaluation of orthopedic physicians [10]. The study by Guermazi et al. describes the development of an AI algorithm capable of interpreting full-size high spatial resolution and multiple radiographic views of the same patient and can be integrated into picture archiving and communications systems [9]. The system has been shown to improve fracture detection sensitivity by 10.4% and is appreciable for all body locations except for shoulder, clavicle and thoracolumbar spine [9]. The AI-based system developed by Lind et al. goes beyond detection and provides detailed fracture classification as by the 2018 AO/Orthopedic Trauma Association (AO-OTA) fracture and dislocation compendium [8]. Results showed a weighted mean AUC of 0.87 for proximal tibia fractures, 0.89 for patella fractures and 0.89 for distal femur fractures. Almost 3/4 of the area under the curve (AUC) estimates were above 0.8, out of which more than half reached an AUC of 0.9 or above [8].

Two studies applied deep learning models to detect cartilage fractures from MRI [7,18]. The study by Wang et al. describes a model that increases clinicians’ accuracy—especially among trainees—for anterior cruciate ligament (ACL) ruptures. The model achieved an area under the receiver operating characteristic curve of 0.987 and a sensitivity and specificity of 95.1% [18]. The study by Chaudhari et al. describes the use of a deep learning super-resolution augmentation algorithm to enhance a 5 min 3D qDESS MRI sequence that automatically generates T2 relaxation time maps [7], increasing sensitivity compared to conventional knee MRI for subtle cartilage lesions. Three studies applied AI to CT scans. Fernandes et al. created an AI algorithm able to convert 2D radiographs to 3D bone model reconstructions with excellent accuracy when comparing all measurement parameters from the CT scan and manual measurements (mean absolute error ≤ 1.98 mm) [17,30,33].

#### 3.4.3. Limb Alignment

Eight papers describe the automatization of the assessment of the of lower-extremity alignment from full leg radiographs (FLRs) and of the calculation of main clinical angles by means of different software architectures and approaches [11,12,13,16,23].

Tack et al.’s study [23] is the first of these to describe a fully automated method for the quantification of hip–knee–ankle (HKA) alignment from FLRs (varus or valgus deformity), using the YOLOv4 And Resnet Landmark regression Algorithm (YARLA). The average deviation of landmarks manually placed by experts and automatically detected by their system was less than 2.0 ± 1.5 mm for all structures [23].

The AI-based system by Erne describes the automatized determination of four parameters in addition to hip–knee–ankle angle (mFAmTA) described by Tack [23], namely mMPTA, mLDFA, mLDTA, and FSAmTA.

Also, the AI-based systems developed by Simon et al. and by Larson et al. provide automatization of clinical angles (mechanical axis HKA and pelvic tilt) as well as additional measurements to those routinely collected with manual annotations, including bone length (femur, tibia, full length), laterality discrepancies, and position of orthopedic hardware; moreover, these systems are also able to generate radiologist-like reports [24,25]. Similarly, Alberti et al. proposed an algorithm for the standardization of Q-angle measurement from whole leg radiographs on a heterogeneous sample of patients, including patients at pediatric age and with bone pathologies [20].

The study by Bernard de Villeneuve et al. [29] described a first in its kind algorithm to help surgeons in preoperatively assessing lower limb deformities and evaluating the appropriate angle for osteotomy.

More recently, Tanner et al. described a detection-based DL algorithm that can detect the bilateral femoral head, knee, and ankle joints with high precision also in patients where the femoral head is difficult to view and calculates HKA angles in LLRs with comparable accuracy to that calculated by manual measurement [36].

Hoffmann et al. [39] tested the performance of a CNN implemented in a common planning software (mediCAD^®^ 7.0; mediCAD Hectec GmbH) that allows analysis and preoperative planning. MediCAD-based analysis demonstrated excellent accuracy for overall lower limb alignment and leg length, but showed significant deviations in joint-level measurements, particularly in cases involving TKA [39].

Finally, Yang J. et al. [35] described a fully automated model that reliably assesses the alignment of lower limbs in patients with knee OA, with or without the presence of a prosthesis, on personal portable devices on LLRs with no need for image pre-processing.

Taken together, results from all the studies above report high accuracy between physician and AI-generated measurements, with similar outcomes in terms of ICC (ICC > 95%) [22,24,25,28,29]. The study by Bernard de Villeneuve et al. features the intra-observer and the interobserver variation on angle determination, which was within 1° [29]. All systems also evidence time saved compared to manual assessment (from 48 to 130 s).

#### 3.4.4. Implant Size

Several studies describe AI-based systems to improve preoperative planning for implant size and positioning compared to conventional X-ray-based templating [33,38,40,41,42]. A few focused on the use of radiographic imaging.

Yu et al. describe a DL-based model using X-ray images alone with no additional information, yielding high predictive power for implant size, especially when using lateral images. The study, however, is based on radiographs from a single surgeon [41]. Park KB et al. [40] compared predictions of an automated preoperative model for TKA on X-rays with the conventional templating by experienced surgeons. Although the model produced only minor differences in terms of accuracy ± 1 levels of the surgeons, it led to a >22% higher agreement of exact accuracy rates for femoral and tibial implant sizes with the actual implant sizes [40]. Other studies described models that did not lead to significant improvements compared to current practice [42].

Others focused on implant description by CT imaging. Li et al. [33] described an AI-based preoperative planning system, AIJOINT, specifically validated for TKA. The system, which includes CT image processing, component planning, and PSI designing modules, requires much less time than traditional CT segmentation (3.74 ± 0.82 vs. 128.88 ± 17.31 min, *p* < 0.05) and PSI design (35.10 ± 3.98 vs. 159.52 ± 17.14 min, *p* < 0.05), and comparable time for size planning. Its accuracy in predicting the size of both femoral and tibial components was 92.9% vs. 42.9% and 47.6%, respectively (*p* < 0.05), of the conventional method in size planning [33]. The study by Liu Z et al., the first DL regression model for automated patellofemoral annotation trained on both physiologic and pathologic CT imaging, reported a mean absolute error between predicted and ground truth landmark coordinates, which was 0.20/0.26 cm in the healthy/arthroplasty cohort [17].

Lan et al. [38] tested an AI-based preoperative 3D planning technology already used in hip implants on prosthesis size and axial alignment planning specifically in TKA and compared it to traditional 2D template measurement technology. The accuracy of prosthesis size, VCA and HKA prediction in the AI group was significantly higher than that in the 2D group (*p* < 0.05). Moreover, WOMAC and AKS scores in the AI group at 3–12 months follow-up after surgery were better than those in the 2D group (*p* < 0.05) [38].

### 3.5. AI Models and Technologies

The reviewed studies highlight the predominance of DL approaches, with only one instance of a ML model [26]. CNNs are the most frequently used architectures, predominantly trained in a supervised fashion [7,10,14,15,17,18,21,22,23,25,27,28,29,31,33,34,35,36,39,41]. Only one study employed self-supervised pretraining, though still followed by supervised fine-tuning [17]. For segmentation tasks, well-established architectures such as UNet and Mask R-CNN dominate [19,22,28,31,33], with only one example of a transformer architecture [42], reflecting the limited adoption of this architecture in knee imaging so far. Interestingly, UNet is also often adopted for landmark localization, framing the position regression (detection) problem into a segmentation one [28,29]. Landmark localization is, however, mostly tackled as an object detection task, for which YOLO [23,36,40] and HRNet [33,35] represent the preferred architectures. RetinaNet was also employed for fracture detection [10] [Liu PR]. In most of these studies, the automated segmentation and/or landmark localization was paramount to the automation of quantitative assessment of alignment parameters [11,14,23,28,29,32,35,36,39] or disease grading [12,19]. AI was also used for classification tasks (e.g., implant classification, patient selection), with ResNet and EfficientNet being the most common AI architectures for this task [8,25,27,41]. Notably, a few studies reported the use of GradCAMs to generate explainability maps in support to ease the interpretation of the outputs of their models [14,21,34]. It is worth also noticing that some commercial AI tools are already present in the landscape and are involved in research studies that assess their usability and robustness, namely LAMA (Leg Angle Measurement Assistant) [11,16,32], KOALA (Knee Osteoarthritis Labeling Assistant) [12], PeekMed [13,30] and RBKnee [19].

Large-scale datasets like the Osteoarthritis Initiative (OAI) play a crucial role in model development, yet validation is often conducted on private cohorts. However, most studies originate from single-center datasets, limiting their generalizability. Notable exceptions are Wang, and Karnuta 2021 and 2023 [18,21,34], which leverage large multicentric cohorts at validation as well as training. Finally, no study explored the development and use of most recent advances with foundation models, indicating a gap in the current research landscape.

## 4. Discussion

The present review aimed to map the recent literature on developments and use of AI tools in diagnostic imaging and preoperative planning of knee joint procedures, identifying the most relevant research studies which have been published up to January 2025. Narrowing the search focus to knee surgery, our analysis identified a total of 36 original studies reporting specific applications of AI in an array of steps, from patient referral and candidate selection to preoperative planning. While in previous years studies prevalently described AI related to X-ray imaging of fracture detection and OA measurement [14], our findings reveal a broader scope of AI applications, especially in the areas of preoperative planning (patient selection and risk assessment) and diagnostic accuracy. Overall, the results of these studies are consistent in reporting higher levels of accuracy gained by applying AI tools to processes that traditionally rely on the specialist’s judgment and activity or on commonly used equipment and software. In practical terms, they highlight the gain in diagnostic accuracy (such as fracture detection and implant characterization) and great potential of streamlining a number of technical tasks, while allowing specialists to focus their attention and time on other activities requiring human agency and supervision. Indeed, in many of the papers published, the authors highlight specific areas where AI-integrated steps would take on a greater role, consequently freeing considerable resources (human, economic, intellectual, time, logistical resources, and so on) for allocation elsewhere. For example, the study by Lind et al. reports an AI-enhanced tool that provides highly accurate measurements to correctly classify fractures by the 2018 AO-OTA fracture classification system and even detect fractures which may be missed by traditional imaging interpretation [8]. The group of Simon et al. [24] found the DL algorithm LAMATM in leg length measurements to match human performance and even exceed it in terms of time and standardization of the technique. Moreover, it produced radiology reports that could potentially replace those drafted by radiologists. Measurement times with AI software were three times faster (saving around 2 min per patient) than manual annotation and were run asynchronously [8]. Finally, the study by Yang J et al. proposed an AI-based system that allows orthopedics to assess limb alignment from their mobile phones based on the availability of X-rays alone, making this extremely attractive for less equipped medical centers [35].

Noteworthy is the steady trend in studies applied to radiographic imaging, which to date remains the most cost-effective and widespread clinical support among most clinical settings.

From a perspective of technological implementation, almost all papers identified are validation studies, where the scope of studies remains narrowed on technical aspects such as efficacy measurements (process accuracy yield, sensitivity, specificity, reliability, replicability, and validity) by means of comparison with retrospective data. Only one specifically involved a prospective deployment of deep learning [17,43]. This represents the first step towards integration of AI technology in routine clinical activity. This should encourage a shift in approach towards its application in daily clinical practice.

No quality improvement assessments nor large-scale studies on clinical practice were found among those screened. Likewise, none covered in detail aspects such as economic evaluations, or studies on patient outcomes, pathways of care, and quality of life. In fact, the set of papers reviewed reflects the current stage of AI implementation, i.e., of ongoing development, technical validation, and testing. As many of these tools become embedded into clinical practice, we expect the types of studies published to shift in focus.

### 4.1. Strengths and Limitations

This review is a first attempt to provide an updated state of the art on the AI-based applications in the field of knee surgery in terms of diagnostic and treatment planning. One aspect worth noting on the findings of this scoping review is the studies being performed on a variety of databases of patient populations from geographical areas which may prevent the generalization of findings as well as a rapid widespread adoption of the algorithms reported.

### 4.2. Next Steps

Most of the studies analyzed in this review are single center and rely on limited validation or test datasets, which hampers the assessment of generalizability and real-world applicability of the proposed AI approaches. Particularly, only a few studies offer evaluations of the training set bias or clarify the range of applicability of their models in accordance with FAIR principles [8,12,18,19,23,31,34]. In relation to this, explainability remains underexplored, with only a handful of studies incorporating tools like GradCAM to aid in interpreting model outcomes [14,21,34]—yet interpretability will be critical for clinical adoption. Finally, some of the presented AI systems are closed source, with limited information on the underlying methodology [12,13,19], making it difficult to compare results across studies or assess the robustness of the approaches. 

Considering these current common limitations, further research and development are necessary for refining AI algorithms, to address challenges related to biased training data, incomplete or unrepresentative datasets, under-representation of populations, implicit assumptions while creating the algorithm, and biased outcomes generated by the algorithm [44]. This involves careful examination and selection of training data, ongoing evaluation of algorithms’ performance, and the implementation of strategies to mitigate and correct biases. The most recent AI modeling strategies have not been fully explored in knee imaging (e.g., transformer-based architectures, large foundation models, vision-language models), leaving a research gap that will be filled in the coming years. However, research should primarily focus on broad spectrum validation of applicability and generalizability of these technologies. In accordance with FAIR principles, AI development should promote transparency and the continual improvement of algorithms by means of studies for validating the effectiveness of algorithms in larger and more diverse patient populations, as well as exploring potential applications beyond imaging and preoperative planning, like robotic-assisted surgery.

## 5. Conclusions

This scoping review shows the recent advances in the field of computer science brought by AI tools in the orthopedic practice, with significant potential for improving diagnostic accuracy, surgical outcomes, and postoperative assessments. As researchers and practitioners continue to explore the possibilities of AI, further advancements and standardization efforts will play a central role in realizing the full potential of AI as a helpful and standardized tool in ordinary practice.

## Figures and Tables

**Table 1:** Papers on AI tools applied to diagnoses of knee conditions.

First Author, Year	Title	Imaging Type	AI Type	AIM/Application	Main Results
Chaudari et al., 2020 [7]	Diagnostic Accuracy of Quantitative Multicontrast 5-Minute Knee MRI Using Prospective Artificial Intelligence Image Quality Enhancement	MRI	Deep Learning	Use DL super-resolution augmentation algorithm to enhance a 5-min 3D qDESS MRI sequence that automatically generates multicontrast images and T2 relaxation time maps	Image quality scores were significantly better for conventional MRI vs. qDRESS for all tissues evaluated (*p* < 0.001), though both methods yielded 92% inter-reader agreement. The qDESS plus T2 method had a higher sensitivity for detecting cartilage lesions (73%) vs. conventional MRI (58%). Automatic T2 relaxation time maps evidenced early chondral degeneration vs. areas that appeared morphologically normal on conventional sequences
Lind et al., 2021 [8]	Artificial intelligence for the classification of fractures around the knee in adults according to the 2018 AO/OTA classification system	X-rays	Convolutional Neural Network	Produce detailed knee fracture classification as by the 2018 AO/Orthopedic Trauma Association (AO-OTA) fracture and dislocation compendium.	A weighted mean AUC of 0.87 for proximal tibia fractures, 0.89 for patella fractures and 0.89 for distal femur fractures. Almost ¾ of AUC estimates were above 0.8, out of which more than half reached an AUC of 0.9 or above.
Guermazi et al., 2021 [9]	Improving Radiographic Fracture Recognition Performance and Efficiency Using Artificial Intelligence	X-rays	Deep Learning	Interpretation of fracture detection full-size high spatial resolution and multiple radiographic views of the same patient for integration into picture archiving and communications systems.	The system has shown to improve fracture detection sensitivity by 10.4% and is appreciable for all body locations except for shoulder, clavicle and thoracolumbar spine. AI assistance improved the sensitivity and may even improve the specificity of fracture detection by radiologists and non-radiologists, without lengthening reading time.
Liu et al., 2021 [10]	Artificial Intelligence to Diagnose Tibial Plateau Fractures: An Intelligent Assistant for Orthopedic Physicians	X-rays	Convolutional Neural Network	Detection of tibial plateau fractures.	The algorithm searched a level of accuracy comparable to orthopedic: 0.91 vs. 0.92 ± 0.03 (*p* = 0.86). The average time spent for analysis of the AI was 16-fold faster than physician: 0.56 s vs. 8.44 ± 3.26 s. However, at the time of the study, the tool was still unable to replace evaluation of orthopedic physicians
Schwarz et al., 2022 [11]	Artificial intelligence enables reliable and standardized measurements of implant alignment in long leg radiographs with total knee arthroplasties	X-rays	Non-specified AI	Evaluation of long leg radiographs (LLR) after total knee arthroplasties (TKA).	AI software was reproducible on 96% and reliable on 92.1% of LLRs with an output and showed excellent reliability in all measured angles (ICC > 0.97) compared to manual measurements. Excellent results were found for primary unconstrained TKA.
Smolle et al., 2022 [12]	Artificial intelligence- based computer- aided system for knee osteoarthritis assessment increases experienced orthopaedic surgeons’ agreement rate and accuracy	X-rays	Convolutional Neural Network	Use of AI-enhanced X-rays by means of the Knee Osteoarthritis Labeling Assistant (KOALA).	Increase in senior reader agreement rates for KL grade (2.0-fold), sclerosis (1.42-fold), JSN (1.37-fold) and osteophyte OARSI grades (3.33-fold); reader specificity and accuracy for all features compared to the gold standard. On the other hand, sensitivity only increased for OA diagnosis. With aided analysis, senior readers reached similar agreement and accuracy rates as junior readers, with both surpassing AI performance.
Yang C. et al., 2023 [13]	In slope-changing osteotomy one millimeter is not one degree: results of an artificial intelligence-automated software analysis	X-rays	Non-specified AI	Investigation of the assumption that 1mm of wedge height gives 1° of slope correction (1:1)	In trans-tubercle anterior closing wedge, using a wedge height (mm) to slope correction (°) ratio of 1:1 can lead to slight over-correction.
Bonnin et al., 2023 [14]	Artificial Intelligence Radiographic Analysis Tool for Total Knee Arthroplasty	X-rays	Convolutional Neural Network	Assistance to surgeons in their postoperative interpretation.	AI tool increased accuracy by 5% and sensitivity by 12% in the detection of interface anomalies. A gain in repeatability for each single surgeon (Light’s kappa 0.17), as well as a gain in reproducibility between surgeons (Light’s kappa +0.1).
Kim MS et al., 2023 [15]	Machine Learning for Detecting Total Knee Arthroplasty Implant Loosening on Plain Radiographs.	X-rays	Convolutional Neural Network	Detection of TKA implant loosening.	The CNN algorithm, through transfer learning, shows high accuracy (sensitivity was 100% and the specificity was 97.5%) for detecting the loosening of TKA implants on plain radiographs
Pagano et al., 2023 [16]	The Role and Efficiency of an AI-Powered Software in the Evaluation of Lower Limb Radiographs before and after Total Knee Arthroplasty	X-rays	LAMA	Assess AI tool efficiency in measuring axial alignments (MAD, AMA), femoral and tibial angles (mLPFA, mLDFA, mMPTA, mLDTA), and other key measurements including JLCA, HKA.	The tool yielded good to excellent agreement with expert metrics (ICC = 0.78–1.00), it analyzed radiographs two-fold faster radiographs twice as fast (*p* < 0.001), yet struggled with accuracy for the JLCA (ICC = 0.79, 95% CI = 0.72–0.84), the Mikulicz line (ICC = 0.78, 95% CI = 0.32–0.90), and if patients had a body mass index higher than 30 kg/m^2^
Liu Z et al., 2023 [17]	Deep Learning for Automated Measurement of Patellofemoral Anatomic Landmarks	CT	Deep Learning	Automatic measurement of patellofemoral anatomy.	Mean absolute error between prediction and ground truth landmark coordinates was 0.20/0.26 cm in the healthy/arthroplasty cohort. There was no statistically significant difference between the model’s predictions and the ground truth measurements for TEA length, TEA-PFA angle, and trochlear medial asymmetry ratio in both cohorts.
Wang DY et al., 2024 [18]	A Deep Learning Model Enhances Clinicians’ Diagnostic Accuracy to More Than 96% for Anterior Cruciate Ligament Ruptures on Magnetic Resonance Imaging	MRI	Deep Learning	Increase clinicians’ accuracy—especially among trainees—for anterior cruciate ligament (ACL) ruptures.	The model achieved an area ROC AUC of 0.987 and a sensitivity and specificity of 95.1%.
Brejnebøl et al., 2024 [19]	Interobserver Agreement and Performance of Concurrent AI Assistance for Radiographic Evaluation of Knee Osteoarthritis	X-rays	Non-specified AI	Improve X-ray knee OA assessment according to the established KL grading system using the AI tool RBknee (version 2.1; Radiobotics).	AI assistance increased junior readers’ radiographic KOA grading performance. Inter-observer agreement for KL grading across radiologists and orthopedics of various experience levels was higher with AI assistance versus without (κ = 0.77 ± 0.018 [SEM] vs. 0.85 ± 0.013; *p* < 0.001). Board-certified radiologists achieved almost perfect agreement for KL grading when assisted by AI (κ = 0.90 ± 0.01).
Alberti et al., 2025 [20]	Artificial intelligence applied to q-angle measurement: preliminary results on an algorithm based on bounding box	X-Rays	Machine Learning with YOLO	Develop an AI-driven algorithm for identification of key points on X-rays for automatic measurement of Q-angle.	Mean prediction errors by algorithm were smallest for the patella: 4.3 (SD ± 2.8) right and 4.0 (SD ± 2.0) left and was highest for the tibial tuberosity 10.3 (SD ± 7.6) right and 8.5 (SD ± 5.7) left.

Abbreviations: AI: artificial intelligence; AUC: area under the curve; CNN: convolutional neural network; KL: Kelgren–Laurence; PFA: posterior femur axis; ROC: receiver operating characteristic curve; SEM: standard error measurement; SD: standard deviation; TEA: transepicondylar axis.

**Table 2:** Papers reviewed on AI tools applied to surgical planning.

First Author, Year	Title	Imaging Type	AI Type	AIM/Application	Main Results
Karnuta et al., 2020 [21]	Artificial Intelligence to Identify Arthroplasty Implants From Radiographs of the Knee	X-rays	Convolutional Neural Network	Implant evaluation	The DL algorithm differentiated between 9 unique knee arthroplasty implants from four manufacturers with near-perfect accuracy.
Schock et al., 2021 [22]	Automated Analysis of Alignment in Long-Leg Radiographs by Using a Fully Automated Support System Based on Artificial Intelligence	X-rays	Convolutional Neural Network	Automatize quantitative analysis of lower-extremity alignment.	ICC ranged from 0.918 to 0.995 (r range, *p* < 0.001), and agreement was almost perfect (intraclass correlation coefficient range, 0.87–0.99). Automatic analysis was faster than the two radiologists’ manual measurements (3 vs. 36 vs. 35 s, *p* < 0.001).
Tack et al., 2021 [23]	Fully automated Assessment of Knee Alignment from Full-Leg X-Rays employing a YOLOv4 And Resnet Landmark regression Algorithm (YARLA): Data from the Osteoarthritis Initiative.	X-rays	YOLO and YARLA	Automatize determination of four parameters in addition to hip–knee–ankle angle from FLRs (varus or valgus deformity	The average deviation of landmarks manually placed by experts and automatically detected by their system was less than 2.0 ± 1.5 mm for all structures.
Simon et al., 2021 [24]	Fully automated deep learning for knee alignment assessment in lower extremity radiographs: a cross-sectional diagnostic study	X-rays	Convolutional Neural Network	Automatize clinical angle measurements of commercially available software of mechanical axis HKA, bone length (femur, tibia, full length), laterality discrepancies, and position of orthopedic hardware.	The system showed good reliability in all lengths and angles (ICC ≥ 0.87) and was 130 s faster than clinicians. Overall accuracy was 89.2% when comparing the AI to the manually measured outputs. sMAD for AI vs. observer was between 0.39 and 2.19° for angles and 1.45–5.00 mm for lengths. It also generates radiologist-like reports.
Larson et al., 2022 [25]	Artificial Intelligence System for Automatic Quantitative Analysis and Radiology Reporting of Leg Length Radiographs	X-rays	Convolutional Neural Network	Quantification of leg lengths/angles and hardware detection from bilateral lower extremity radiographs using a keypoint-based artificial intelligence system.	AI hardware detection demonstrated an accuracy of 99.8%. Automatic quantitative and qualitative analysis of leg length radiographs using deep learning is feasible and holds potential in improving radiologist workflow.
Lambrechts et al., 2022 [26]	Artificial Intelligence Based Patient-Specific Preoperative Planning Algorithm for Total Knee Arthroplasty	N.A.	Machine Learning	Implant size prediction	The average number of corrections a surgeon has to make to the preoperative plan generated using AI was reduced by 39.7% compared to the manufacturer’s default plan.
Houserman et al., 2022 [27]	The Viability of an Artificial Intelligence/Machine Learning Prediction Model to Determine Candidates for Knee Arthroplasty	X-rays	Deep Learning	Assess the viability of a knee arthroplasty prediction model using 3-view X-rays (3 different X-ray views (anterior–posterior, lateral, and sunrise) that helps determine if patients with knee pain are candidates for TKA, UKA.	Achieved an accuracy of 87.8% on the holdout test set and a quadratic Cohen’s kappa score of 0.811. The multiclass receiver operating characteristic area under curve score for TKA was calculated to be 0.97; for UKA a score of 0.96 and for No Surgery a score of 0.98 was achieved. An accuracy of 93.8% was achieved for predicting Surgery versus No Surgery and 88% for TKA versus non TKA.
Erne et al., 2022 [28]	Automated Artificial Intelligence-Based Assessment of Lower Limb Alignment Validated on Weight-Bearing Pre- and Postoperative Full-Leg Radiographs	X-rays	Convolutional Neural Network	Determine the mMPTA, mLDFA, mLDTA, and FSAmTA	The ICC values of human vs. AI inter-rater reliability analysis ranged between 0.8 and 1.0 preoperatively and between 0.83 and 0.99 postoperatively (all *p* < 0.001).
Bernard de Villeneuve et al., 2022 [29]	An artificial intelligence based on a convolutional neural network allows a precise analysis of the alignment of the lower limb	X-rays	Convolutional Neural Network	Aid surgeons in assessing the preoperative of lower limb deformities and evaluating the appropriate angle for osteotomy	The algorithm showed high accuracy for automated angle measurement, allowing the estimation of limb frontal alignment to the nearest degree.
Fernandes et al., 2022 [30]	Accuracy, Reliability, and Repeatability of a Novel Artificial Intelligence Algorithm Converting Two-Dimensional Radiographs to Three-Dimensional Bone Models for Total Knee Arthroplasty	X-rays and CT scans	Non-specified AI	Convert 2D radiographs to 3D bone models.	A high degree of accuracy, reliability, and repeatability for converting 2D radiographs to 3D bone reconstructions similarly to a CT-scan. Mean absolute errors were <2 mm in 9/12 anatomical parameters vs. measurements performed on CTs and manual calipers. All inter-observer and intra-observer correlation coeficients were > 0.90.
Steele et al., 2023 [31]	Deep Learning Phenotype Automation and Cohort Analyses of 1946 Knees Using the Coronal Plane Alignment of the Knee Classification	X-rays	Deep Learning	Automated knee phenotyping and analyzed CPAK parameters (including the lateral distal femoral, medial proximal tibia, hip–knee–ankle, and joint line obliquity angles).	No significant difference in the CPAK angles (n = 140, *p* = 0.66–0.98, ICC = 0.89–0.91) or phenotype classifications made by the algorithm and surgeon (*p* = 0.96). Women had more valgus CPAK phenotypes than men (*p* < 0.05). Patients who had higher KL grades at baseline (2 to 4) were more varus using the CPAK classification compared to lower KL grades (0 to 1) (*p* < 0.05).
Huber et al., 2023 [32]	Gender-specific distribution of knee morphology according to CPAK and functional phenotype classification: analysis of 8739 osteoarthritic knees prior to total knee arthroplasty using artificial intelligence	X-rays	LAMA	Analyze the preoperative knee morphology with regard to CPAK and functional phenotype in LLR prior to TKA surgery	Distribution in knee morphology with gender-specific differences highlights the wide range in osteoarthritic knees, characterized by CPAK and phenotype classification and may influence future surgical planning
Li S et al., 2023 [33]	Development and Validation of an Artificial Intelligence Preoperative Planning and Patient-Specific Instrumentation System for Total Knee Arthroplasty	CT	NEURAL NETWORK	Develop the AI preoperative planning and PSI system (AIJOINT) for TKA and validate its time savings and accuracy in clinical applications	AIJOINT accurately predicted the component size planning the size of femoral and tibial components was 92.9%, while the accuracy of the conventional method in planning the size of the femoral and tibial components was 42.9% and 47.6%, respectively (*p* < 0.05). It significantly reduced the time needed for CT processing and PSI design without increasing the time for size planning.
Karnuta et al., 2023 [34]	Artificial Intelligence for Automated Implant Identification in Knee Arthroplasty: A Multicenter External Validation Study Exceeding 3.5 Million Plain Radiographs	X-rays	Deep Learning	External validation of knee arthroplasty classification systems	The model discriminated nine implant models with an AUC of 0.99, accuracy 99%, sensitivity of 95%, and specificity of 99% in the external-testing dataset. Excellent internal and external validation, suggesting immediate global scale use in assisting in preoperative planning prior to revision knee arthroplasty.
Yang J et al., 2024 [35]	“Automatic measurement of lower limb alignment in portable devices based on deep learning for knee osteoarthritis”	X-rays	Non-specified AI	Lower limb alignment analysis with portable devices (mobile phones) from X-rays.	In both the validation and test sets, the average mean radial errors were 2.778 and 2.447 (*p* < 0.05). The test results for native knee joints showed that 92.22%, 79.38%, 87.94%, 79.82%, and 80.16% of the joints reached angle deviation < 1° for HKA, JCLA, AMA, mLDFA, and mMPTA. Additionally, for joints with prostheses, 90.14%, 93.66%, 86.62%, 83.80%, and 85.92% of the joints reached that.
Tanner et al., 2024 [36]	Developing a Computer Vision Model to Automate Quantitative Measurement of Hip-Knee-Ankle Angle in Total Hip and Knee Arthroplasty Patients	X-rays	Deep Learning	Calculation of HKAA in THA and TKA patients and assessed the agreement between DL-derived HKAAs and manual measurement.	The algorithm could detect the bilateral femoral head, knee, and ankle joints with high precision, even where the femoral head is difficult to visualize. The inter-rater reliability between manual and DL-derived HKAA measurements on the operative leg and nonoperative leg indicated excellent reliability (ICC (2,k) = 0.987 [0.96, 0.99], intraclass correlation (2,k) = 0.987 [0.98, 0.99, respectively]).
Tandel J et al., 2024 [37]	Evaluating axial alignment and knee phenotypes in a young Indian population, using X-rays converted to 3D bone models, and their relevance in total knee arthroplasty	X-rays	Non-specified AI	Quantify the percentage of the young healthy Indian population have a HKA = 180° and the percentage of this population with deviation from the neutral HKA.	A significant portion of the normal population had limbs that deviated from neutral HKA.
Lan et al., 2024 [38]	Reliable prediction of implant size and axial alignment in AI-based 3D preoperative planning for total knee arthroplasty	X-rays	Neural Network	Assess 3D planning technology on prosthesis size and axial alignment planning in TKA vs. 2D X-ray template measurement.	The accuracy of prosthesis size, VCA and HKA prediction in AI group was significantly higher with AI-based preoperative 3D planning technique than that in 2D group (*p* < 0.05).
Hoffmann et al., 2024 [39]	High accuracy in lower limb alignment analysis using convolutional neural networks, with improvements needed for joint-level metrics	X-rays	Convolutional Neural Network	Implementation of a common planning software (mediCAD^®^ 7.0; mediCAD Hectec GmbH) that allows analysis and preoperative planning.	mediCAD-based analysis demonstrated excellent accuracy for overall lower limb alignment and leg length, but showed significant deviations in joint-level measurements, particularly in cases involving TKA. CNN evaluation demonstrated high consistency in measuring leg length (ICC > 0.99) and lower limb alignment measures of mTFA (ICC > 0.97; RMSE < 1.1°). The mean absolute difference between angular measurements were low for lower limb alignment (mTFA 0.49–0.61°) and high for specific joint angles (aMPFA 3.86–4.50°).
Park KB et al., 2024 [40]	Clinical validation of a deep learning-based approach for preoperative decision-making in implant size for total knee arthroplasty	X-rays	Deep Learning	Predict implant sizes based on automatized measurement of the femoral and tibial regions without manual annotation.	Higher agreement levels achieved by the proposed DL model demonstrate its potential as a valuable tool in preoperative decision-making for TKA. Exact accuracies for predicting femoral and tibial implant sizes were 61.54% and 68.38% by surgeon and 89.32% and 90.60% by DL model.
Yu Y et al., 2024 [41]	Development of an artificial intelligence model for predicting implant size in total knee arthroplasty using simple X-ray images	X-rays	RESNET	Predict implant size using X-ray images alone without any other data (such as demographics), to achieve a model with strong predictive power.	The model showed micro F1-score 0.91 for femur and 0.87 for tibia. For predicting within ± 1 size, micro F1-score was 0.99 for femur and 0.98 for tibia.
Park J et al., 2024 [42]	A deep learning based automatic two-dimensional digital templating model for total knee arthroplasty	X-rays	Non-specified AI	Automatization of implant size prediction.	Exact predictions for 39.5% of fem- oral and 43.2% of tibial components. Allowing a one-size margin of error, 88.9% of predictions were deemed “accurate” for both components.

Abbreviations: CT: computed tomography; AMA: anatomical mechanical angle; CPAK: coronal plane alignment of the knee; HKA: hip–knee angle; HKAA: hip–knee–ankle angle; ICC: intraclass correlation coefficient; MAD: mechanical axis deviation; mLPFA: femoral and tibial angles mLDFA: mechanical lateral distal femoral angle; mLDTA: mechanical lateral distal tibia angle; mMPTA: mechanical medial proximal tibia angle; mechanical tibio-femoral angle (mTFA); TKA: total knee arthroplasty; VCA: valgus correction angle.

## Data Availability

The original contribution presented in this study is included in the article.
